# Evaluation of horizontal ridge augmentation using beta tricalcium phosphate and demineralized bone matrix: A comparative study

**DOI:** 10.4317/jced.51244

**Published:** 2013-12-01

**Authors:** Mahmoud A. Shalash, Hatem A. Rahman, Amr A. Azim, Amani H. Neemat, Hesham E. Hawary, Sherine A. Nasry

**Affiliations:** 1 Researcher, Department of Oral Surgery & Medicine, National Research Center, Giza, Egypt; 2 Professor, Department of Oral & Maxillofacial Surgery, Faculty of Oral and Dental medicine, Cairo, Egypt; 3 Professor, Department of Oral Radiology, Faculty of oral and dental medicine, Cairo, Egypt; 4 Professor, Department of Oral Surgery & Medicine, National Research Center, Giza, Egypt; 5 Lecturer, Department of Oral & Maxillofacial Surgery, Faculty of Oral and Dental medicine, Cairo, Egypt; 6 Researcher, Department of Oral Surgery & Medicine, National Research Center, Giza, Egypt

## Abstract

Objectives: To evaluate the effectiveness of beta tricalcium phosphate (β-TCP) alone compared to β-TCP and Demineralized Bone Matrix (DBM) in regenerating localized horizontal maxillary alveolar ridge deficiencies prior to implant placement. Study Design: The study included 20 patients with horizontal maxillary ridge deficiencies limited to one or more neighbouring teeth and initial ridge width of ≤ 5mmm. Patients were divided equally into two equal groups. Ridge augmentation was performed using Guided Bone Regeneration (GBR) principals. In group I GBR was performed using β-TCP only, while in group II both β-TCP and DBM were used. Following a 6 months healing period, bone cores from both groups were retrieved and implants were inserted. Specimens were examined histologically to calculate percentage of mineralized bone. Apical and crestal changes in ridge dimensions were calculated by digital subtraction using Cone Beam Computed Tomography (CBCT) immediately after graft placement and six months later. Results: There was a statistically significant difference between the mean area percentage of mineralized bone between both groups where it was 40.1 % (range: 27.76-% 66.29 %) for group I and 68.96 % (range: 60.07 % - 87.33 %) for group II. Radiograpically, the mean ridge width in group I increased crestally to 4.66 mm (range:3.5-5mm) and apically to 6.12 mm (range: 4.1-6.7 mm). In group II the mean ridge width increased crestally to 5.2 mm (range 4.9-5.4mm) and apically to 6.9 mm (range 6.0-7.8 mm). Group II showed more bone gain with a mean of 1.37 mm crestally and 2.44 mm apically. This difference however was not statistically significant Conclusion: Within the limitations of this study the combination of DBM and β-TCP can be used effectively in cases exhibiting minimal alveolar ridge defects.

** Key words:**Guided bone regeneration, equine bone, alloplast, bone graft.

## Introduction

Alveolar bone loss can occur after tooth extraction, trauma, as a result of advanced periodontal disease or failed endodontic therapy (1). If the alveolar ridge is not preserved at the time of tooth extraction or loss, alveolar ridge height and width may be lost, particularly in the area of the facial plate. Several system reviews have reported losses between 3 & 6 mm horizontally and 2 mm vertically (2).

An essential condition for successful implant therapy is the presence of an adequate quantity and quality of bone. A significant problem, however, is insufficient height and width of the alveolar bone at the implantation site (3). Several methods have been developed to augment the deficient bone volume. These include Guided Bone Regeneration (GBR) with barrier membranes, onlay bone grafting, ridge splitting and distraction osteogensis (4).

Although the use of autogenous bone has been widely accepted as the gold standard augmentation material; intra-and extra-oral donor site morbidity, potential complications and risks associated with the harvesting procedures have been reported. Because of these drawbacks most recent GBR research has focused on augmentation procedures using bone substitution materials of allogenic, alloplastic, or xenogenic origin in combination with barrier membranes (5).

Cerasorb M® represents a new generation of pure phase beta tricalcium phosphate (β-TCP). The material is highly biocompatible and provides a unique interconnecting scaffold that allows it to be completely resorbed while simultaneously facilitating new bone formation (6). The material is sintered prior to packaging and has an intragranular porosity of 65%. This granulates (polygonal morsals) have a particle size ranging from 150-500um and 500-1000um (7). With its consistent porosity and calcium phosphate ratio, the material provides predictable resorption and new bone formation within 4-12 months. The capillary effect of the blood within Cerasorb M ® granules promotes the rapid formation of osteoblasts which stimulates vital bone growth (8).

Activagen ® (Osteoplant activagen ®, Biotek, Italy) is a material composed of lyophilized granules of type I collagen prepared from a xenogenic equine source and has been described as an activator of morphogenesis. The product is prepared by enzymatic deantigenation of equine femurs bones. The process eliminates all the organic components of bone (proteins, lipids & sugars) except collagen type I .As the material becomes implanted in the graft site, it undergoes degradation in the presence of osteoclasts. The lyophilized protein factors become released, undergo hydration and, are able to exercise the morphogenetic effect on the undifferentiated mesenchymal cell elements, inducing their differentiation into osteoblasts leading to the formation of new bone tissue (9).

## Material and Methods

- Patients’ selection 

20 patients (12 females and 8 males), age range from 18 years to 45 years (average 31.5 years) underwent GBR using non-resorbable barrier membranes, followed by implant placement after six months. All patients had localized horizontal alveolar ridge deficiency, limited to one or two neighbouring teeth, with an initial ridge width of ≤ 5 mm. and 21 potential implant sites. This was determined preoperatively using a ridge mapping caliber (Oraltronics, Bremen, Germany), and Cone Beam Computed Tomograhy (CBCT). Subjects with medical conditions or lifestyle factors likely to affect healing were excluded from the study, including those with uncontrolled diabetes, immune disease, history of alcohol or drug abuse, current smokers, or individuals deemed to be a compliance risk.The protocol of this study was approved by the Ethical Committee of the National Research Center, Egypt. The selected patients were divided into two equal groups; Group I , where GBR was performed using β-TCP, (Cerasorb M ®,Cursan AG, Germany), and group II where Cerasorb M ®, was mixed with DBM (Osteoplant activagen ®, Biotek, Italy) in a 1:1 ratio according to the manufacturer recommendations.

- Surgical and grafting procedure

All patients received prophylactic antibiotic (1 gm. Amoxicillin-clavulanate, Augmentin ®, GlaxoSmithKline, UK) one hour preoperatively and then every twelve hours for four days and were instructed to rinse with 0.12% Chlorhexidine gluconate (Lysoplaque ®, France) for one minute prior to surgery and twice daily for 2 weeks postoperatively. All procedures were performed under local anathesia utilizing articaine hydrocholoride 4% with 1:100000 epinephrine (Septanest ®, Septodont, USA). Full thickness periosteal flaps were elevated. A drill was used to make a channel in the deepest part of the bony defect to anchor a 1.3 mm micro bone screw (Biomet microfixation, Florida, USA).

Following screw insertion the bone surface was decorticated to stimulate bleeding from the marrow compart-ment. In group I the β-TCP was prepared according to the manufacturer’s recommendations where it was mixed with the patient’s own blood. For patients in group II both the β-TCP and DBM were mixed together before adding the patient’s blood. Following adaptation of the graft material, a high density poly-tetrafluroethyelene membrane (Cytoplast GBR-200, Oraltronics, Bremen,Germany) was applied and fixed in place using titanium bone tacks (Dentium, Seoul, S. Korea). The flaps were adjusted using periosteal releasing incisions to provide tension free closure and flaps were sutured using 3-0 silk sutures (Ethicon, Johnson & Johnson, France).

Augmentin (1gm tablets) was prescribed for the patients to be taken regularly every 12 hours for 4 days. Diclofenac Sodium 75 mg intramuscular injection (Voltaren, Novartis, Switzerland) was given every 12 hours for the first 48 hours and then was replaced with 50 mg enteric coated tablets taken only when needed. Patients were instructed to rinse with 0.12% Chlorohexidine gluconate 3 times per day for two weeks. Sutures were removed 15 days following the surgery. Patients were followed up for signs and symptoms of pain, inflammation, infection, wound dehiscence or membrane exposure 24 hours postoperatively, then day after day for the first week, and then monthly for 6 months.

- Radiographic Evaluation

An immediate CB CT scan was taken for each patient to serve as a reference point for future measurements. Following a six months healing period, another CBCT scan was performed to evaluate the outcomes of the regeneration process.

Analysis of the changes in ridge width was made on both the CBCT software and image J software. CBCT images of the area of interest were generated by redrawing the dental arches using identical reference points. After generating the panoramic view, Serial cuts of 1.5 mm thickness were obtained in the sagittal plan. Pre and post-operative images having the same reference number of bony cut were then selected. For each case; the initial CBCT scan and the corresponding 6 CBCT scan were superimposed using Image J software and the amount of change at the crest and 10 mm apical to the crest were calculated by digital subtraction 

- Sample Collection and Implant Procedure

Following 6 months healing period, a flab was elevated the membrane and tentative screw were removed and the area was irrigated was copious saline. A bone core biopsy was taken from the implant sites with a 2 mm diameter trephine drill (Hu-Friedy, USA). The osteotomy was further enlarged using twist drills, following the regular sequence of drilling recommended by the manufacturer and a suitable implant (Screwplant, Implant direct LLC, USA) was screwed in position to the desired depth. Fixtures were covered with the covering screw followed by copious amount of irrigation and the flaps were re-approximated using tension-free interrupted sutures and post-operative instructions were given

- Histomorphometric analysis 

Bone biopsies were fixed in 10% neutral buffered formaldehyde for two weeks. Specimens were decalcified in Ethylene Dimethyl Tetra Acetic Acid (EDTA). The solution was changed daily for one week. Decalcification was considered complete when the specimens reached a rubber like consistency. The specimens were then dehydrated in ascending grades of alcohol (50%, 75%, and 100%), cleared in xyline and then embedded in paraffin. The paraffin-embedded specimens were serially cut in a cross sectional plane into sections of five micron thickness and mounted on glass slides. The sections were de-paraffinized, hydrated, and stained with haematoxylin and eosin.

Sections were examined with (10X magnification) using Olympus CX20 microscope (Olympus Europa Holding GmbH, Hamburg), attached to a camera and computer. All the stained sections were examined by image analyzer computer system using the ImageJ software (NIH, version v1.45e, USA) capable of performing high speed digital image processing for the purpose of tissue measurements.Image J software was calibrated and the images were opened on the computer screen for pre-analysis adjustments.

**Figure 1:**
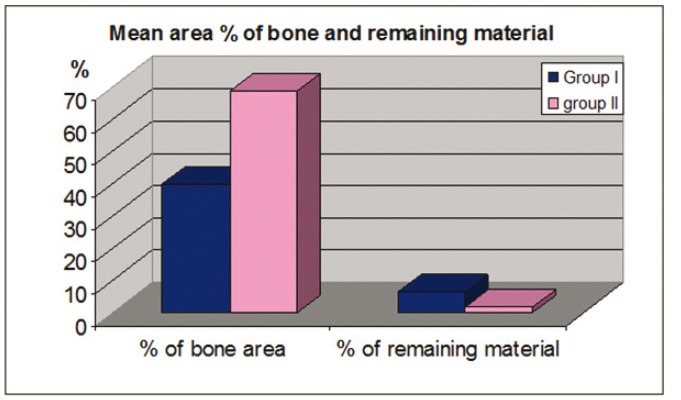
Histogram demonstrating the mean bone gain of ridge width apically and crestally for Group I and Group II.

**Figure 2:**
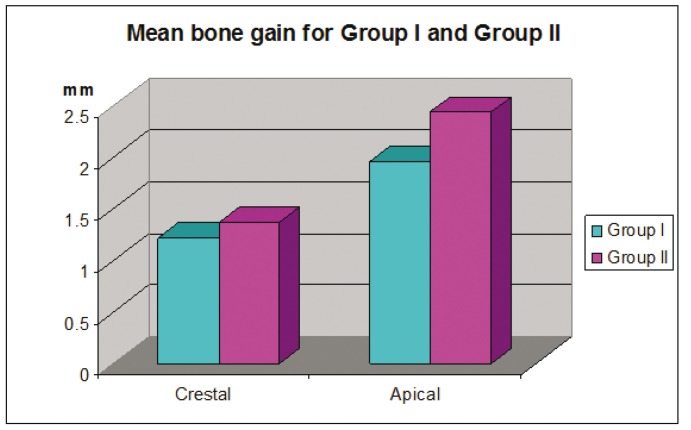
Histogram showing the mean area percentage of mineralized bone and remaining graft material in Group I and Group II.

Histomorphometric measurements were used to quantify the relative amounts of different tissue types within the grafted area. The following variables were measured: area percentage of mineralized bone and area percentage of remaining graft particles when found. Mean values for histomorphometric variables were calculated for each group. For histomorphometric analysis of the area percent of bone, the color of bone trabeculae was automatically selected, converted into grey then masked by a red color which allowed automatic measurement by the computer system. (Fig. 1,2).

- Statistical Analysis

Statistical analysis was performed using SPSS (Statistical package for the social sciences) (version 15, Echosoft Corp, U.S.A.) Data were represented as mean ± standard deviation. Paired sample student t-test was used to compare each pair of the studied variables within the studied group of patients. Independent sample t-test was used to compare variables between the two studied groups. The test result was considered statistically significant if the P value was equal to or less than 0.05.

## Results

- Clinical Findings

One case from each group demonstrated early exposure of the barrier membrane which required their removal after four weeks leaving only 18 patients with 19 potential implant sites. All remaining cases healed uneventfully without any serious adverse effects and demonstrated clinically significant increase in alveolar ridge width, and resistance to drilling when the implant bed was being prepared.

- Histological findings 

In group I, the remaining graft material was seen as spherical structures with various forms and sizes. Mineralized bone was seen between and surrounding the remaining graft particles. Evidence of osteoblastic rimming and osteoid tissue having viable osteocytes were seen in close proximity to the mineralized bone suggesting active bone formation.The general findings showed evidence of remodeling and new bone surrounding the graft fragments. In group II, large amounts of mineralized bone tissue with minimal traces of the graft material surrounded by newly formed bone tissue were consistently seen. Evidence of osteoblastic rimming and osteoid tissue were seen in close proximity to the mineralized bone suggesting active bone formation .

## Statistical analysis

- Histologically

Analysis of the histological sections demonstrated that the mean area percentage of mineralized bone was 40.1 % (range: 27.76-% 66.29 %) for group I and 68.96 % (range: 60.07 % - 87.33 %) for group II. The mean area percentage of remaining β-TCP was 6.5 % (range: 3.36 -10.63 %) for group I and 1.7 % (range: 0.15%- 4.5 %) for group II ( Table 1). There was a statistically significant difference between both groups, where group II had a higher area percentage of mineralized bone and a smaller area percentage of the remaining graft material than group I ( Table 2).

**Table 1:**
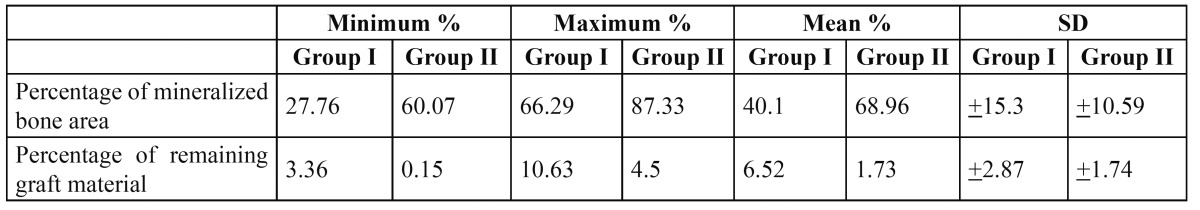
Means and standard deviations for the area percentage of mineralized bone and remaining graft material in Group I and Group II.

**Table 2:**
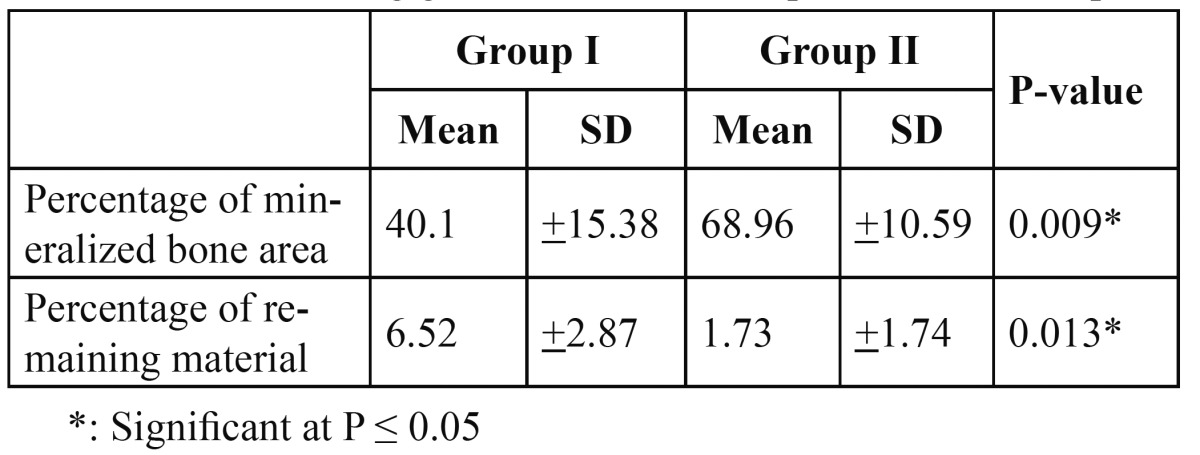
Comparison between the mean area percentage of mineralized bone and remaining graft material of Group I & that of Group II.

- Radiographically

For group I, the mean ridge width apically in the initial CBCT was 4.1 mm (range: 2.0-3.6 mm), while crestally it was 3.44 mm (range: 1.7-5mm). Analysis of the CBCT scan taken 6 months later showed that the mean ridge width apically increased to 6.12 mm (range: 4.1-6.7 mm) and crestally to 4.66 (range: 3.5-5 mm) ( Table 3). In group II, the mean ridge width apically in the initial CBCT was 4.5 mm (range 3.8-5mm), while crestally it was 3.8 mm (range 3.3-4.3mm). Postoperative analysis of the CBCT scan 6 months later showed that the mean ridge width apically was increased to 6.9 mm (range 6.0-7.8 mm) and crestally to 5.2 mm (range 4.9-5.4mm) ( Table 3).

**Table 3:**
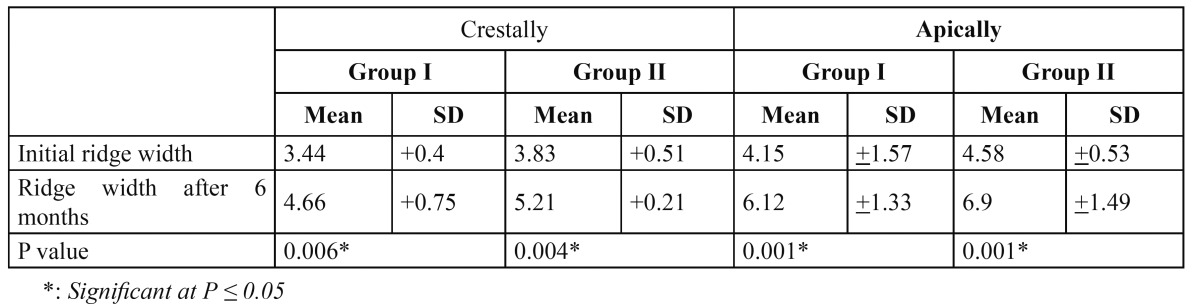
Means and Standard deviations for the alveolar ridge widths (initial and 6 months later) in Group I and II.

The amount of bone gain both apically and crestally was statistically significant for both groups. Although group II showed more bone gain with a mean of 1.37 mm crestally and 2.44 mm apically, this difference was not statistically significant ( Table 4).

**Table 4:**
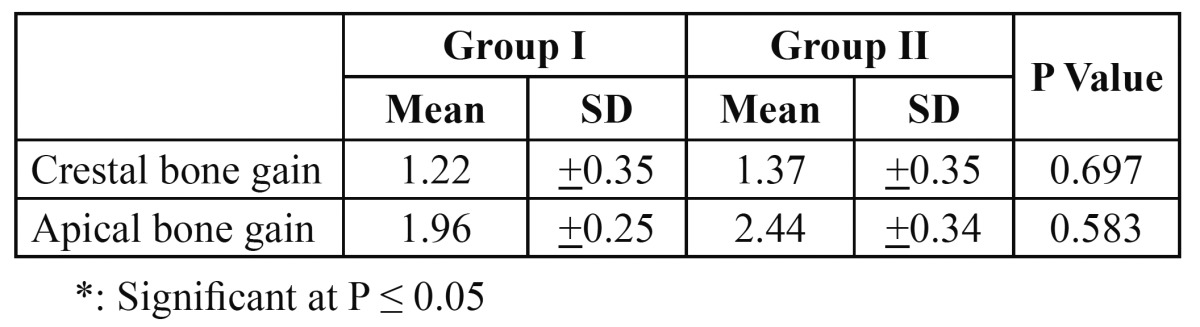
Comparison between the bone gain apically and crestally of Group I and that of Group II.

## Discussion

This study evaluated the radiographic and histological outcomes of localized ridge augmentation utilizing DBM of equine origin (Activagen)® combined with β–TCP (Cerasorb M)® compared to β–TCP alone. Non resorbable barrier membranes were chosen owing to their better space-maintaining abilities, controlled time of barrier function, lack of a resorption process and higher percentage of bone regeneration that can be achieved with their use compared to resorbable barriers (10).

One case in each group experienced failure of the bone graft to augment the alveolar ridge to an ideal dimension suitable for implant placement. This was due to dehiscence of the flaps and exposure of the membranes which required their early removal. Several studies have demonstrated membrane exposure to be the most common postoperative complication following GBR (11,12).

One of the possible causes for early membrane exposure could be attributed to necrosis of a thin flap covering the membrane. In GBR, blood supply to the flaps depends on flap thickness because blood supply from the bone to the flap is impeded by the membrane (13).Another possible cause for early membrane exposure could be insufficient release of horizontal periosteal incisions of the buccal flap that led to tension and subsequent ischemia of the tissues at the suture line. In order to prevent this it was recommended that the buccal and lingual/palatal flaps should overlap by at least 10 mm to obtain a tension-free closure (14).

Cerasorb M was chosen for horizontal ridge augmentation owing to its biocompatibility and satisfactory enhancement of bone regeneration as reported by several studies (15,16).

In the present study mineralized bone was seen between and surrounding the remaining graft particles with evidence of osteoblastic rimming in close proximity to the mineral bone suggesting active bone formation. The results are in accordance with previous reports (17,8) where bone and osteoid tissue were seen lying in contact with the remaining β–TCP granules suggesting that these granules attract osteoprogenitor cells that migrate into the micropores of the graft material . Histological results of both groups demonstrating minimal traces of β-TCP are in accordance with those of other studies which have shown that the material can be replaced completely by new bone within a six months healing period (18,19).

Results of this study demonstrated higher percentage of new bone formation and lesser percentage of remaining graft material in group II than group I. This is in agreement with the histomorphemetric analysis of a previous study that showed that the rate of breakdown of the graft material was proportional to the amount of new bone regeneration (20).

DBM has been shown to induce osteoblastic differentiation and proliferation of bone marrow mesenchymal stem cells. Histologic evidence of osteoblasts penetrating the surface of the DBM granules denoted the material’s ability to support osteoblastic cell adhesion (21). Results showed that the density of the newly formed bone inside surgically created gaps filled with DBM reached 93% of native bone density after 12 weeks (22). Moreover radiographic evidence of new bone formation after using injectable DBM during sinus elevation surgery was seen following a 6 months healing period (23).

With the material’s ability to induce differentiation of mesenchymal cells to form bone as well as serve as a scaffold for osteoprogenitor cells attachment (22,24,25), the combined effect of adding both DBM and β-TCP to augment the deficient horizontal alveolar ridge was evaluated in this study.

To our knowledge no other human studies compared the effect of using of β-TCP alone versus β-TCP and DBM in regenerating alveolar ridge deficiencies.

One study evaluated the osteogenic activity of β-TCP and DBM in a rabbit model, where osseous defects were surgically created on the right and left edentulous ridges of the mandibles and one side filled with the DBM and the other with β-TCP. Histological results showed that by the end of the 6 week mature bone tissue had completely filled the defects. The DBM was completely resorbed at 5 weeks; however remnants of the β-TCP were still present (25). In contrast to these findings another research used DBM for regenerating dehiscence defects around dental implants and found that remnants of the material were still present six months following grafting. However the authors were able to demonstrate that new bone formation and graft resorption occurred as healing progressed (26). When comparing the histological results for both groups it was found that there was a statistically significant difference between the amount of mineralized bone and remnants of the graft material in group II (68.9% & 1.2 % respectively) compared to group I (40.1% & 6.5%) (26).

For all patients in both groups there was a significant increase in ridge width along its entire length, relative to the pre-surgical values of each group. Although the gain in ridge width measured on the CBCT for group II was found to be more than in the control, the difference was not found to be statistically significant when both groups were compared.

Bone formation in grafts is thought to occur via three mechanisms of bone deposition: 1) Osteogenesis, osteoinduction, and osteoconduction, in which the graft acts as a scaffold for deposition of new bone by adjacent living bone (27). β-TCP bone substitute used in this study works by osteoconduction, i.e. by guiding osteogenic cells from existing bone. These osteogenic cells differentiate into osteoblasts, which form bone between the separate graft particles 34.

DBM works through both osteoinduction and osteoconduction. The material induces osteoblasts’ and chondroblasts’ differentiation from mesenchymal cells. With its osteoinductive properties DBM leads to an increase in the number of available osteoblasts at the graft site (26). The presence of β-TCP which supports osteoblasts adhesion (28), combined with DBM at the defect site, can explain the higher percentage of mineralized bone that was found in the test group and more bone gain that was measured radiographically.

The outcome of this study is comparable to former studies that used xenografts alone or in combination with allogenic DBM to regenerate horizontal ridge defects. These results are also in agreement with evidence from the literature that both materials have the ability to support new bone formation (29,30).

## Conclusion

GBR using d-PTFE membranes and an alloplast (β-TCP) can result in successful horizontal ridge augmentation sufficient for placement of endosseous implants. Combining both β-TCP with DBM can further enhance the amount of bone gain in the defect area. Further investigations and a larger study sample are required to verify the outcomes of this research.
